# Nickel toxicity alters growth patterns and induces oxidative stress response in sweetpotato

**DOI:** 10.3389/fpls.2022.1054924

**Published:** 2022-11-10

**Authors:** Sunjeet Kumar, Mengzhao Wang, Yi Liu, Shah Fahad, Abdul Qayyum, Sultan Akbar Jadoon, Yanli Chen, Guopeng Zhu

**Affiliations:** ^1^ Key Laboratory for Quality Regulation of Tropical Horticultural Crops of Hainan Province, School of Horticulture, Hainan University, Haikou, China; ^2^ Sanya Nanfan Research Institute, Hainan University, Sanya, China; ^3^ Department of Agronomy, Abdul Wali Khan University Mardan, Mardan, Pakistan; ^4^ Department of Agronomy, The University of Haripur, Haripur, Pakistan; ^5^ Department of Plant Breeding and Genetics, The University of Agriculture, Peshawar, Pakistan

**Keywords:** nickel, sweetpotato, oxidative stress, antioxidant defense system, gas exchange, photosynthetic pigments

## Abstract

Nickel (Ni) contaminated soil is a persistent risk to plant growth and production worldwide. Therefore, to explore the Ni toxicity levels in sweetpotato production areas, we investigated the influence of different Ni treatments (0, 7.5, 15, 30, and 60 mg L^-1^) for 15 days on phenotype, Ni uptake, relative water content, gas exchange, photosynthetic pigments, oxidative stress, osmolytes, antioxidants, and enzymes of sweetpotato plants. The results presented that Ni at higher levels (30 and 60 mg L^-1^) substantially reduced growth, biomass, and root morphological traits. The Pearson correlation analysis suggested that Ni toxicity causes oxidative injuries as persistent augmentation of hydrogen peroxide (H_2_O_2_) and malonaldehyde (MDA) and reduced RWC, gas exchange, and photosynthetic pigment. Furthermore, this study revealed that sweetpotato could tolerate moderate Ni treatment (up to 15 mg L^-1^) by reducing oxidative stress. The results also indicated that the increase in the activities of mentioned osmolytes, antioxidants, and enzymes is not sufficient to overcome the higher Ni toxicity. Based on these results, we suggest using low Ni-contaminated soil for better growth of sweetpotato and also could be used as a phytoremediator in moderate Ni-contaminated soil.

## Introduction

Heavy metals are the critical problem for crops, animals, and humans. However, a minute amount of heavy metals can help enhance plants’ growth and production (Amjad et al., 2019). Such as, nickel (Ni) is a heavy metal and a vital microelement for the growth and yield of plants, and it is an irreplaceable primary part of urease enzymes (Kumar et al., 2015; Zhao et al., 2019). The deficiency of Ni can restrict urease activity, which leads to necrosis in the leaf tips (Fabiano et al., 2015). The average concentration of Ni in plants usually ranges from 0.05 - 10 mg kg^–1^, but higher concentrations display toxicity symptoms in plants (Yusuf et al., 2011). However, in the last few decades, Ni concentration in the soil and water has increased due to different anthropogenic activities, including industries, smelting, municipal sludge, combustion, electroplating, and fertilizers, which is a severe threat to humans health (He et al., 2011; Xu et al., 2011; Zhao et al., 2019). According to Yusuf et al. (2011) and Sreekanth et al. (2013), nickel (Ni) concentrations in polluted soils range from 200–26,000 mg kg^−1^. Ni has affected the agricultural land in central and south China, and the average Ni concentration was 226.30 mg kg^−1^. The highest concentration of Ni was 1000 mg kg^−1^ in the agricultural land of Gansu Province, which is significantly higher than the permissible limits (35.2 mg kg^−1^) (Rizwan et al., 2017).

Although a minute quantity of Ni is essential for growth, it turns toxic for plants at higher concentrations (Gajewska and Skłodowska, 2005). Previous studies showed that higher Ni concentrations had induced phytotoxicity by reducing reduced the growth and relative water content and disrupting the photosynthetic assimilation, pigments, enzymatic activity, and osmolytes, which lift chlorosis and necrosis of the leaf (He et al., 2012; Awasthi and Sinha, 2013; Fourati et al., 2019). Furthermore, plants accumulate heavy metals primarily in the roots, then translocate them to the shoots. Previous studies revealed that some plants use their roots as the prime storage organ for heavy metals, whereas others exhibited toxicity tolerance in shoots (Yang et al., 2010; Fahad et al., 2015). Ni application in higher concentrations can also interrupt cellular homeostasis by overproduction of reactive oxygen species (ROS), which ultimately cause oxidative damage to morphological, anatomical, and ultrastructure structures of the plant leaves and roots (Tappero et al., 2007; Gajewska and Skłodowska, 2008; Israr et al., 2011; Fourati et al., 2019). Previous studies reported that elevated Ni significantly increased many plants’ hydrogen peroxide and lipid peroxidation (Uruç Parlak, 2016; Fourati et al., 2019; Khair et al., 2020).

However, plants have sophisticated antioxidant defense mechanisms to counter oxidative stress and scavenge ROS, which include antioxidant enzymes (superoxide dismutase (SOD; EC 1.15.1.1), peroxidase (POD; EC 1.11.1.7), catalase (CAT; EC 1.11.1.6), and ascorbate peroxidase (APX; EC 1.11.1.1), antioxidants (carotenoids, glutathione (GSH), polyphenols, and flavonoids), and osmolytes (glycine betaine, proline, soluble sugars, and total proteins) (Michalak, 2006; Kumar et al., 2015; Amjad et al., 2019; Kumar et al.,2020). Different plants respond differently to Ni treatments and plant some plants are more sensitive to Ni than others and show deleterious affected the physiological and metabolic processes of several plants. Their findings also revealed that Ni toxicity depends on the plant genotypes, cultivation methods, growth stage, exposure time, and chemical form of Ni (Khellaf and Zerdaoui, 2010; Yusuf et al., 2011; Zhao et al., 2019).

Sweetpotato (*Ipomoea batatas* L.) is a rich starch source and is the 6^th^ most important crop globally (Park et al., 2020). Apart from storage roots, the leaves and stems are used as green vegetables in different countries (Shekhar et al., 2015; Fu et al., 2016). Sweetpotato leaves are a rich source of valuable nutrients, such as vitamins, dietary fibers, minerals, protein, carotenoids, and total polyphenols (Ishiguro et al., 2007; Kurata et al., 2007; Sun et al., 2014). Sweetpotato is tolerant to temperature and drought, but heavy metals can severely affect its growth and productivity (Mwanga et al., 2017). Few studies showed that a minor quantity of Ni might improve the growth and production of plants (Singh et al., 2004; Gajewska and Skłodowska, 2008). In contrast, several studies showed that Ni application hindered the metabolic processes of different plants and showed a deleterious effect on their growth (Uruç Parlak, 2016; Ameen et al., 2019; Amjad et al., 2019; Fourati et al., 2019). Different studies described deviations in response to Ni stress, and toxicity and tolerance level also varied in different plants. Ni toxicity and tolerance level vary within plant species, genotypes, and growth stages. There have been few studies on the negative effects of Ni in sweetpotato up to this point. Furthermore, no detailed information on the antioxidant defense system of Ni toxicity in sweetpotato is available. Therefore, it was necessary to emphasize the effects of Ni stress on sweetpotato plants, particularly to determine the Ni-toxicity level in sweetpotato. In this regard, the current study was designed to investigate the tolerance level of sweetpotato plants under Ni stress. We investigated the effects of different levels of Ni stress on sweetpotato growth, photosynthesis, oxidative stress, osmolytes, antioxidants, and enzymes.

## Materials and methods

### Seedling growth and treatment

The sweetpotato cultivar “Haida HD7791” was used in this experiment. Sweet potato cuttings were disinfected with 1 g L^-1^ Carbendazim for 5-8 min and grown in Ro water until roots appeared. After that, The cuttings were acclimated in half Hoagland nutrient solution (pH 5.8 ± 1) for a few days (Hoagland and Arnon, 1950). Hydroponic experiments were conducted in a controlled environment (25-27°C, 16-h photoperiod) to assess the Ni effect on sweetpotato plants. The nutrient solution was changed every 5 to 6 days to ensure proper nutrition. Seedlings of nearly uniform size and health were chosen and divided into five treatment groups. Ni was used in the dosages of 0, 7.5, 15, 30, and 60 mg/L as Nickel chloride hexahydrate (NiCl_2_·6H_2_O) for 15 days. After 15 days of Ni stress, samples were collected for further analysis.

### Growth parameters

After 15 days, the plant height, number of leaves, leaf area, shoot and root fresh (FW), and dry weight (DW) were measured. The topmost leaves were used for the leaf area determination, and the data were collected using a portable laser leaf area meter (CI-202). The FW of the shoots and roots of the plants were calculated. After that, the shoots and roots were placed in the incubator at 70°C for 2 days to determine the DW (Kumar et al., 2021b). For shoot dry weight susceptibility index (SDSI) and root dry weight susceptibility index (RDSI) calculation, the following formula reported by Qayyum et al. (2021) was used.


(1) $$SDSI=\frac{Shoot\;DW\;(Stressed\;Plants)}{Shoot\;DW\;(Controlled\;Plants)}\times 100$$

(2) $$RDSI=\frac{Root\;DW\;(Stressed\;Plants)}{Root\;DW\;(Controlled\;Plants)}\times 100$$

### Relative water content analysis

A protocol reported by Kumar et al. (2021b) was used to determine the relative water content (RWC). The FW of the leaves was recorded first, then the leaves were immersed in ddH_2_O for 4 h in a Petri dish, and the turgor weight of the immersed leaves was recorded. After that, the leaves were placed in the incubator at 70°C for 24 h to determine their dry weight. Finally, the RWC of the leaves was calculated using the following formula:


(3) RWC% = [(FW - DW)/(TW - DW)] × 100

### Gas exchange parameters and root morphology

For the determination of gas exchange parameters, completely developed leaves were analyzed using a portable photosynthesis system (CIRAS-3, Hansatech Co., USA) (Altaf et al., 2020). The roots of each plant were collected and washed with distilled water. Then, the roots were scanned with the help of the Imagery Scan Screen (Epson Expression 11000XL, Canada), and for the determination of root traits, WinRHIZO 2003a software was used (Altaf et al., 2020).

### Chlorophyll measurement

Around 100 mg of fresh leaves were homogenized with 80% acetone using a glass homogenizer. Then, the homogenized samples were centrifuged at 8,000 × *g* for 15 minutes and collected supernatant. A Full wavelength microplate reader (Infinite M200 PRO, TECAN, Swiss) was used to measure the absorbance of chlorophyll a, b, and carotenoids at 663, 646, and 470 nm, respectively (Kumar et al., 2021a). The concentration was determined with the following formula:


(4) Chl a= 12.21(A663) - 2.81(A646)

*Chl* *b*= 20.13(*A*646)–5.03(*A*663) (5)


(6) Car = [1000(A470) – 3.27(chl a) - 104(chl b)]/229

### Determination of MDA, H_2_O_2_, proteins, GSH, and antioxidant enzymes

About 100 mg of fresh leaves were homogenized with 0.9 mL of 0.1 M phosphate buffer saline (PBS) (pH 7.4). The homogenized samples were centrifuged at 5,000 × *g* for 15 min. The collected supernatant was utilized for the MDA quantification with the help of a kit (A003-1-1) purchased from Nanjing Jiancheng Bioengineering Institute, Nanjing, China. Finally, its absorbance was recorded at 530 nm (Kumar et al., 2021b).

About 0.5 g of fresh leaves were homogenized adequately with 4.5 mL of 0.1 M PBS; the homogenate were centrifuged at 10,000 × *g* for 15 min. The collected supernatant was used to determine the content of H_2_O_2_, total proteins, GSH, and antioxidant enzymes (CAT, POD, SOD, and APX) with the kits purchased from Nanjing Jiancheng Bioengineering Institute, Nanjing, China. The total protein content was determined using the Coomassie brilliant blue method with a commercially available total protein assay kit (A045-2), and the absorbance was measured at 595 nm. H_2_O_2_ forms a complex with molybdate, whose absorbance at 405 nm was measured. The glutathione assay kit (A006-1) was used to determine the GSH content using the DTNB [5,5’-dithiobis (2-nitrobenzoic acid)] method. GSH content was expressed as mg g^−1^ protein, and absorbance was measured at 420 nm. The APX assay kit (A123-1-1) was used to determine the APX activity. APX catalyzed ascorbate oxidation at 290 nm and expressed as U mg^−1^ protein. Using the SOD assay kit (A001-1), the activity of SOD was calculated and displayed as U mg^−1^ protein. POD activity was measured using a POD assay kit (A084-3-1) based on guaiacol oxidation at 470 nm by H_2_O_2_ and expressed as U mg^−1^ protein. Similarly, CAT activity was determined using a CAT assay kit (A007-1) and expressed as U mg^−1^ protein (Yang et al., 2018; Kumar et al., 2020; Kumar et al., 2021b).

### Determination of proline and soluble sugars

For the determination of proline, an assay kit (A107-1-1) was used. About 0.1 g of fresh leaves were homogenized with the company’s buffer and followed the company’s protocol; finally, its absorption value was determined at 520 nm. Approximately 50 mg of fresh leaves were properly homogenized for the soluble sugars in 0.45 mL ddH_2_O using a glass homogenizer. The homogenate were kept at 95°C for 15 min then the tubes were cooled. Then, the homogenate was centrifuged for 15 min at 7,500 × *g*, and the supernatant was collected. The supernatant was diluted 1:9 with ddH_2_O. A commercial test kit measured soluble sugar in the diluted extracts (A145-1-1). Finally, its absorbance was recorded at 620 nm and the results were expressed on the fresh weight (FW) basis (Kumar et al., 2020; Kumar et al., 2021b).

### Determination of total polyphenols and flavonoid content

The protocol of Kumar et al. was used to quantify total polyphenols and flavonoids (Kumar et al., 2021a; Kumar et al., 2022). About 1 g of fresh leaf samples were crushed and homogenized with 60% ethanol. About 1.25 mL of 10% Folin–Ciocalteu reagent, 0.25 mL plant extract, and 1 mL sodium carbonate decahydrate solution (0.75 g/mL) were mixed. The mixture was incubated for about 15 minutes at 45°C and then retained at room temperature for half an hour. Finally, its absorbance was recorded at 765 nm, and the data was presented against the gallic acid (GAE/g) standard.

For flavonoid content, about 0.25 mL of NaNO_2_ (0.5 g/mL) solution was mixed with 2 mL ddH_2_O and 500 µL of extract. The mixture was placed at 27°C for 5 min. Then 150 µL of aluminum chloride solution (1 g/mL), 1 mL of sodium hydroxide (1 M) solution, and 1.2 mL of ddH_2_O were added concurrently. Finally, its absorbance was recorded at 510 nm, and the data was presented against the catechin (CAE/g) standard.

### Ni content analysis

The concentration of Ni was measured using the wet digestion method. After seven days of treatment, the plants were cut, rinsed with Ro water, and dried to constant weight. The 100 mg powdered plant samples were digested with 2 ml HNO_3_, 0.5 ml H_2_O_2_, and 1 ml deionized water using a super microwave-assessed digestion system (Anton Paar, Multiwave 7000, AUSTRIA). The content of Ni in the prepared samples was determined by ICP-MS (Perkin Elmer, NexION 5000G, USA) (Kumar et al., 2021b). The following formula was used to calculate the uptake and translocation of Ni.


(7) Ni uptake (mg) = tissues Ni concentration × tissues dry mass

(8) $$Root\;to\;shoot\;Ni\;translocation\;=\frac{concentration\;of\;Ni\;in\;the\;shoots}{concentration\;of\;Ni\;in\;the\;roots}$$

### Statistical analysis

All the phenotypic, physiological, and biochemical experiments were executed in triplicates. We used SPSS 25.0 software, and Duncan tests were applied for the determination of significant differences (*p* ≤ 0.05) among the Ni-treated and control groups, and these differences were presented with different alphabets in the figures. GraphPad Prism 7 (San Diego, California, United States) was used for the figures. All the study results are represented as mean ± standard error (S.E). Principal component analysis (PCA) and Pearson correlations were performed using the “ggplot2” package in R (version 3.3.4, https://CRAN.R-project.org/package=ggplot2).

## Results

### Plant phenotypic parameters, susceptibility index, and RWC

**Figure 1:**
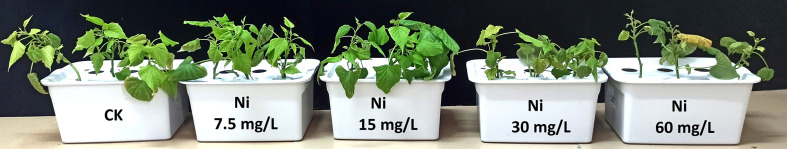
Effect of different Ni treatments on the sweetpotato growth.

**Figure 2:**
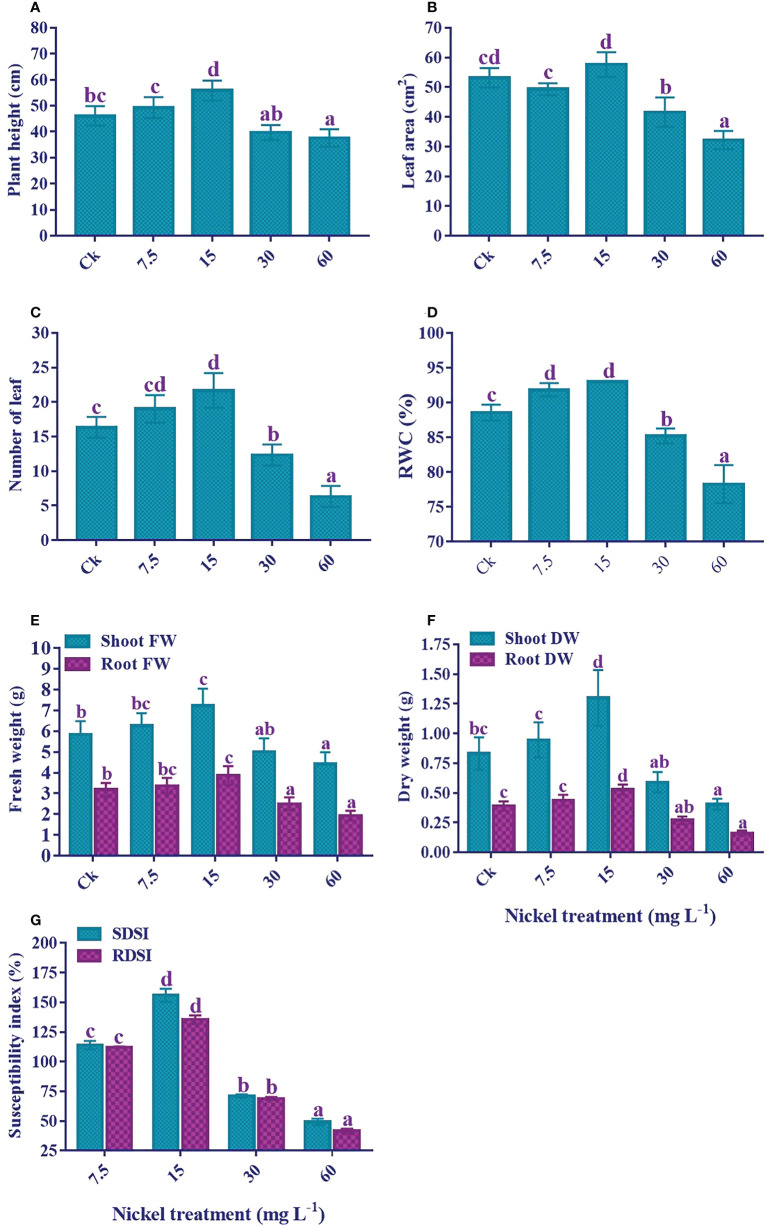
Effect of different Ni treatments on growth, biomass, and susceptibility index of sweetpotato. **(A)** Plant height, **(B)** Leaf area, **(C)** Number of leaf, **(D)** relative water content, **(E)** fresh weight of the plant, **(F)** dry weight of the plant, and **(G)** shoot and root dry weight susceptibility index. According to the Duncan test, different letters indicate a significant difference (p < 0.05) among the five treatments. The error bars represent the mean ± SE.

Ni treatment significantly influenced the growth of sweetpotato (**Figure 1**). The plant treated with 7.5 and 15 mg L^-1^ Ni showed an increase in the growth traits; plant height, leaf area and number, RWC, shoot and root FW and DW, SDSI, and RDSI; however, a significant decrease in these parameters was observed at higher concentrations (30 and 60 mg L^-1^) (*p*< 0.05; **Figure 2**). As compared to the control, the plant height (7% and 21.1%), number of leaves (16.3% and 32.6%), shoot FW (7% and 23.6%), root FW (6.1% and 21.5%), shoot DW (13.9% and 56.3%), root DW (12.3% and 35.2%), SDSI (14% and 56%), and RDSI (12.3% and 35.4%) were increased at 7.5 and 15 mg L^-1^ Ni treatment, respectively. Interestingly, leaf area under 7.5 mg L^-1^ Ni treatment showed a decrease of 7.2%, but at 15 mg L^-1^ Ni treatment increase of 8.4% was detected (**Figure 2B**). Likewise, RWC in the leaves also increased significantly at 7.5 and 15 mg L^-1^ Ni treatment (**Figure 2D**). Conversely, plant growth revealed a negative correlation at the higher concentrations of Ni treatment. Furthermore, a maximum reduction was observed at 60 mg L-1 Ni treatment, with reductions in plant height, leaf area, leaf number, shoot and root FW and DW, SDSI, and RDSI of 18.5%, 39.5%, 61.2%, 24.4%, 39.3%, 51.2%, 58.5%, 50.8%, and 58.5% in comparison to control, respectively. RWC followed the same pattern and showed an 11.6% decrease under 60 mg L^-1^ Ni treatment compared to the control (**Figures 2A–G**).

### Root growth traits

**Figure 3:**
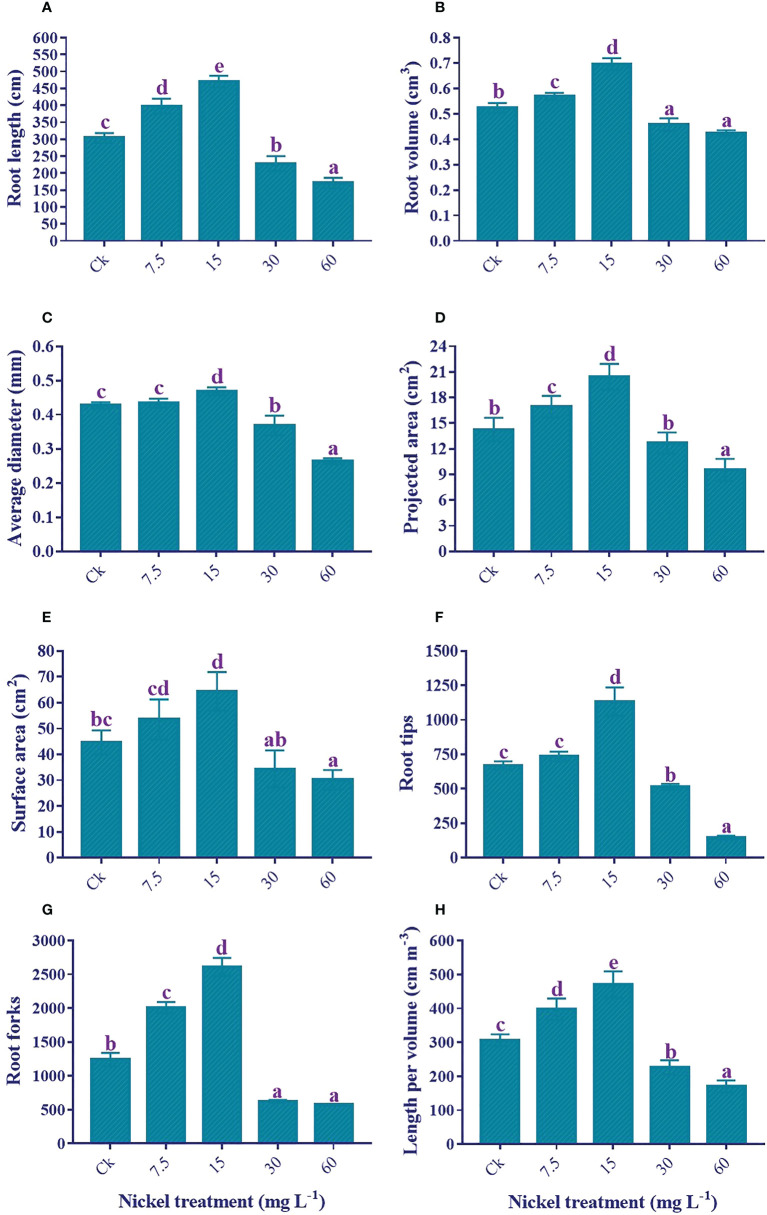
Effect of different Ni treatments on root morphology of sweetpotato; **(A)** root length, **(B)** root volume, **(C)** surface area, **(D)** average diameter, **(E)** projected area, **(F)** root tips, **(G)** root forks, and **(H)** root length per volume. According to the Duncan test, different letters indicate a significant difference (*p*< 0.05) among the five treatments. The error bars represent the mean ± SE.

Ni treatment significantly influenced the root growth traits. Sweetpotato plants treated with 7.5 and 15 mg L^−1^ Ni presented an increase in the root growth traits, and the highest increment was detected at 15 mg L^−1^ Ni treatment (**Figure 3**). The root length (54.6%), root volume (32.8%), surface area (44.1%), average diameter (9.8%), projected area (43.2%), tips (69.1%), crossing (17.1%), forks (110.1%), and length per volume (53.7%) were increased with the treatment of 15 mg L^−1^ Ni in comparison to the control. Conversely, higher concentrations of Ni (30 and 60 mg L^−1^) treatment exhibited a substantial reduction in the root morphological traits, and a maximum reduction was detected at 60 mg L^−1^ Ni treatment (**Figure 3**). The maximum reduction in comparison to control was 43.8%, 18.9%, 32.6%, 38.1%, 33.1%, 78.5%, 53.8%, and 44.3% in the root length, total root volume, total average diameter, projected area, surface area, tips, forks, and length per volume, respectively (**Figures 3A–H**).

### Leaf gas exchange analysis

**Figure 4:**
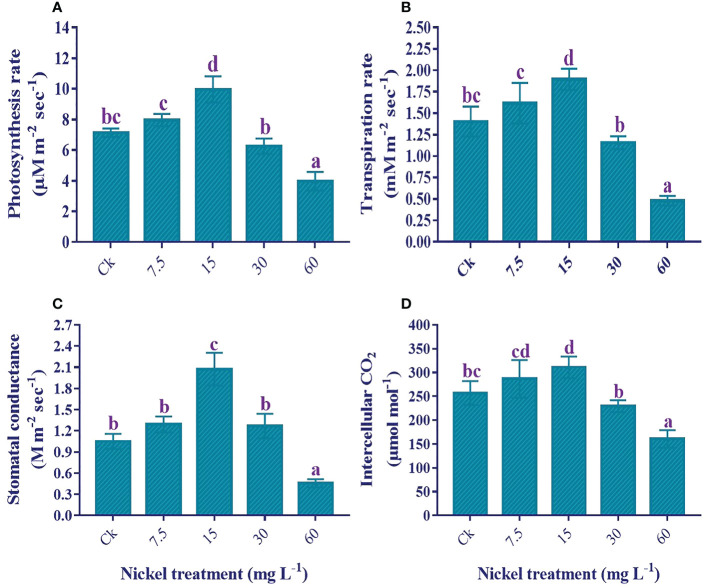
Effect of different Ni treatments on gaseous exchange elements in the leaf of sweetpotato; **(A)** photosynthetic rate (Pn), **(B)** transpiration rate (Tr), **(C)** stomatal conductance (Gs), and **(D)** Intercellular CO_2_ (Ci). According to the Duncan test, different letters indicate a significant difference (*p*< 0.05) among the five treatments. The error bars represent the mean ± SE.

The application of low Ni increased leaf gas exchange elements; however, higher applications of Ni (30 and 60 mg L^−1^) had an adverse effect on leaf gas exchange elements in comparison to the control (**Figure 4**). Furthermore, a maximum reduction was found in 60 mg L^−1^ Ni-treated plants. As compared to the control, photosynthesis rate (11.6% and 39.5%), transpiration rate (15.2% and 35.1%), stomatal conductance (22.8% and 97.4), and intercellular CO_2_ (11.5% and 21%) were increased at 7.5 and 15 mg L^-1^ Ni treatment, respectively (**Figure 4**). On the other hand, maximum reductions of 44.4%, 65.7%, 56.5%, and 37.5% were noticed in the Pn, Tr, Gs, and Ci, respectively, compared to the control (**Figure 4**).

### Photosynthetic pigments

**Figure 5:**
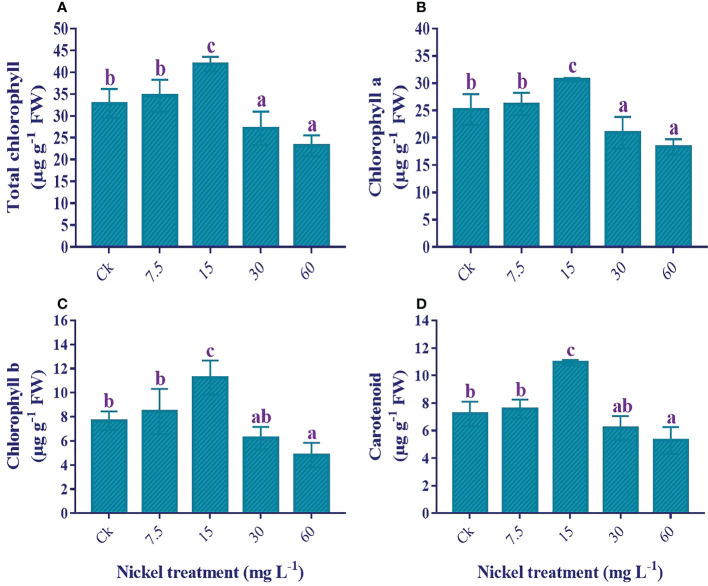
Influence of different Ni treatments on the photosynthetic pigments in the leaves of sweetpotato. **(A)** total chlorophyll, **(B)** chlorophyll a, **(C)** chlorophyll b, and **(D)** carotenoids. According to the Duncan test, different letters indicate a significant difference (*p*< 0.05) among the five treatments. The error bars represent the mean ± SE.

The application of Ni stress significantly influenced the photosynthetic pigments. Under 7.5 and 15 mg L^−1^ Ni treatment, we found an increase in total chlorophyll (T. chl), chl a, chl b, and carotenoids, with the highest content found in 60 mg L^−1^; 27.6%, 21.8%, 46.6%, and 51.5% increase in T. chl, chl a, chl b, and carotenoids contents in comparison to the control. In contrast, 30 and 60 mg L^−1^ Ni treatments presented a significant decrease in the photosynthetic pigments, and the maximum decrease was observed in 60 mg L^−1^. Compared to the control, 29.5%, 27.1%, 37.1%, and 26.7% reductions in the content of T. chl, chl a, chl b, and carotenoids were observed (**Figure 5**).

### H_2_O_2_ and MDA content determination

**Figure 6:**
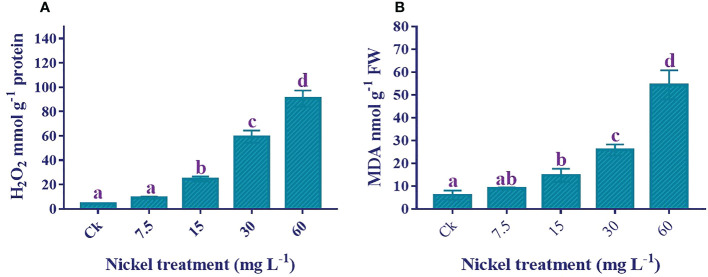
Effect of different Ni treatments on the H_2_O_2_ and MDA in the leaves of sweetpotato. **(A)** H_2_O_2_ content and **(B)** MDA content. According to the Duncan test, different letters indicate a significant difference (p < 0.05) among the five treatments. The error bars represent the mean ± SE.

Lipid peroxidation and reactive oxygen species production increased stress under Ni treatment. H_2_O_2_ and MDA contents were significantly triggered by Ni treatment (**Figure 6**). Applying Ni stress increased MDA and H_2_O_2_ contents, and the highest H_2_O_2_ and MDA contents were detected in 60 mg L^−1^ Ni treatment. The H_2_O_2_ and MDA contents in 60 mg L^−1^ Ni-treated plants were 2064.6% and 808.6% higher than the control.

### Osmolytes production

**Figure 7:**
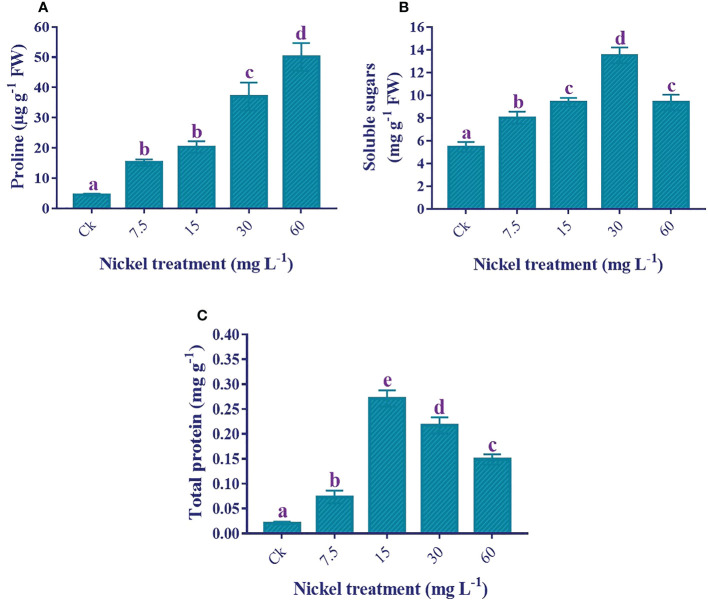
Effect of different Ni treatments on the production of osmolytes in the leaves of sweetpotato. **(A)** proline content, **(B)** soluble sugar content, and **(C)** total protein content. According to the Duncan test, different letters indicate a significant difference (*p*< 0.05) among the five treatments. The error bars represent the mean ± SE.

Osmolytes production was increased to overcome Ni stress. The application of Ni treatments significantly enhanced the proline production *(p*< 0.05), and the maximum increase of 1013.6% in proline production was recorded at 60 mg L^−1^ Ni treatment in comparison to the control (**Figure 7**). Likewise, soluble sugar production was also considerably higher in Ni-treated plants. The soluble sugars content was increased to 30 mg L^−1^, whereas the soluble sugar content decreased at 60 mg L^−1^ but was still 72.1% higher than the control (**Figure 7**). Similarly, the total protein content increased to 15 mg L^−1^, then decreased at 30 and 60 mg L^−1^ but was still significantly higher than the control and 7.5 mg L^−1^ Ni treatment (**Figure 7C**).

### Antioxidant enzymes activities

**Figure 8:**
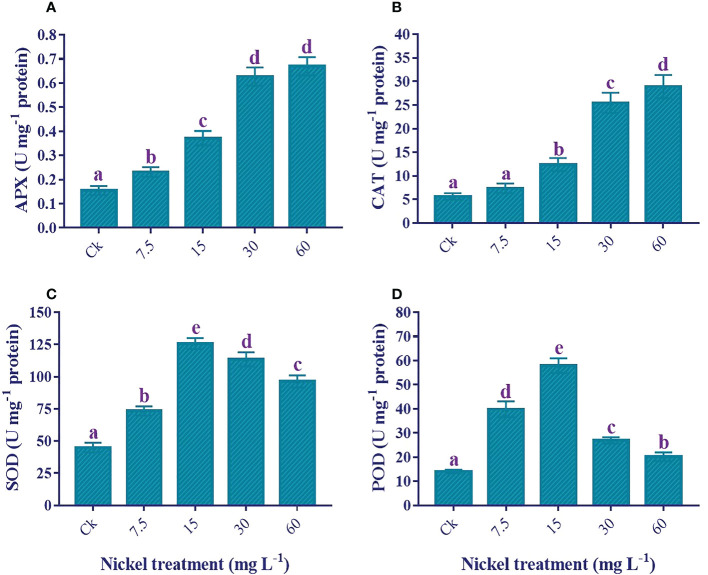
Effect of different Ni treatments on the antioxidant enzymes in the leaves of sweetpotato. **(A)** APX, **(B)** CAT, **(C)** SOD, and **(D)** POD. According to the Duncan test, different letters indicate a significant difference (*p*< 0.05) among the five treatments. The error bars represent the mean ± SE.

An increase in antioxidant enzyme activities plays a role in overcoming Ni stress, and Ni treatments significantly influence enzymatic activities (*p*< 0.05). The APX and CAT were positively influenced by Ni treatment, and the highest level was found at 60 mg L^−1^ Ni treatment (**Figures 8A, B**). Furthermore, 333.2% and 409.6% increments were detected in the APX and CAT, respectively, compared to the control. Similarly, the SOD and POD activities increased to 15 mg L^−1^, then reduced at 30 and 60 mg L^−1^ Ni treatment, but the activities of both enzymes were still significantly higher than the control (**Figures 8C, D**). Moreover, plants treated with 60 mg L^-1^ Ni depicted a 114.3% increase in the activity of SOD and a 43.5% increase in the activity of POD compared to the control.

### Antioxidants analysis

**Figure 9:**
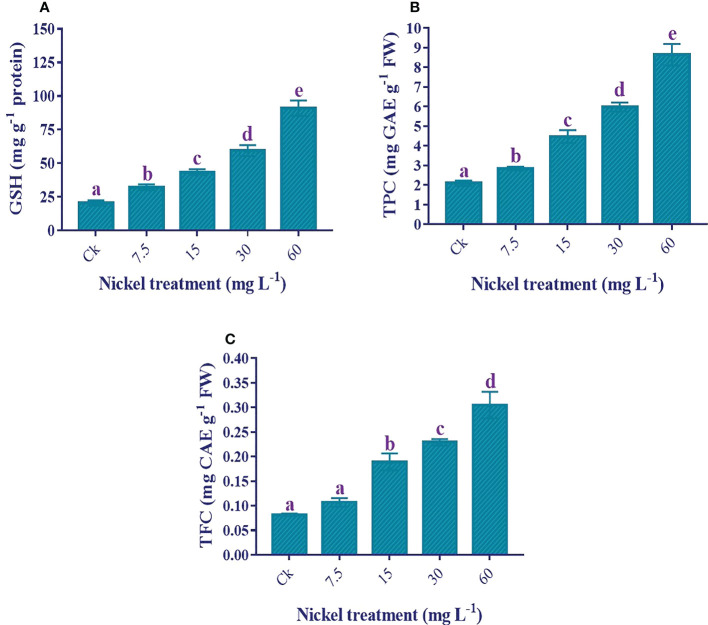
Effect of different Ni treatments on the antioxidants in the leaves of sweetpotato. **(A)** GSH, **(B)** total polyphenols, and **(C)** total flavonoids. According to the Duncan test, different letters indicate a significant difference (*p*< 0.05) among the five treatments. The error bars represent the mean ± SE.

An increase in antioxidants helps to overcome Ni stress. The GSH, total polyphenols, and total flavonoid contents were increased with Ni treatment, and the highest level was found at 60 mg L^−1^ Ni treatment (**Figures 9A–C**). Furthermore, the maximum increment of 344.2%, 312.7%, and 273.5% under 60 mg L^−1^ Ni treatment was detected in the content of GSH, total polyphenols, and total flavonoids, respectively, compared to the control (**Figures 9A–C**).

### Nickel determination

**Figure 10:**
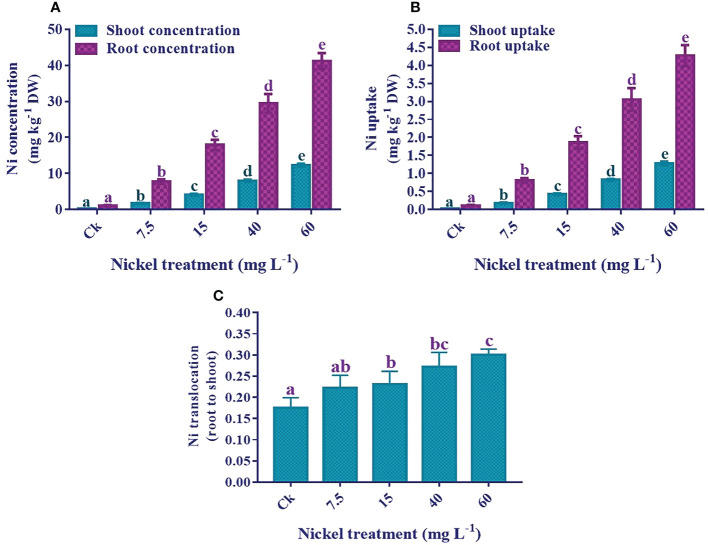
Effect of different Ni treatments on the concentrations, uptake, and translocation (root to shoot) of Ni in the sweetpotato. **(A)** Ni concentration in shoots and roots, **(B)** Ni uptake, and **(C)** Ni translocation. According to the Duncan test, different letters indicate a significant difference (*p*< 0.05) among the five treatments. The error bars represent the mean ± SE.

The results showed that an increase in Ni application significantly elevated the Ni concentration and uptake in both shoots and roots. The highest Ni concentration and uptake were recorded in 60 mg L^−1^ treated plants (**Figure 10**). Furthermore, the Ni accumulation in the roots was higher than in the sweetpotato shoots. Ni concentration was 12.4 mg kg^−1^ DW in the shoots and 41.2 mg kg^−1^ DW in the 60 mg L^−1^ treated roots. Moreover, Ni uptake by shoots and roots significantly increased with the increase of the Ni level, and maximum uptake was 1.3 mg kg^-1^ DW in the shoots and 4.3 mg kg^-1^ DW in the roots. Similarly, the Ni translocation was also significantly increased with the increase of Ni application (**Figure 10**).

### Correlation coefficient and principal component analysis

**Figure 11:**
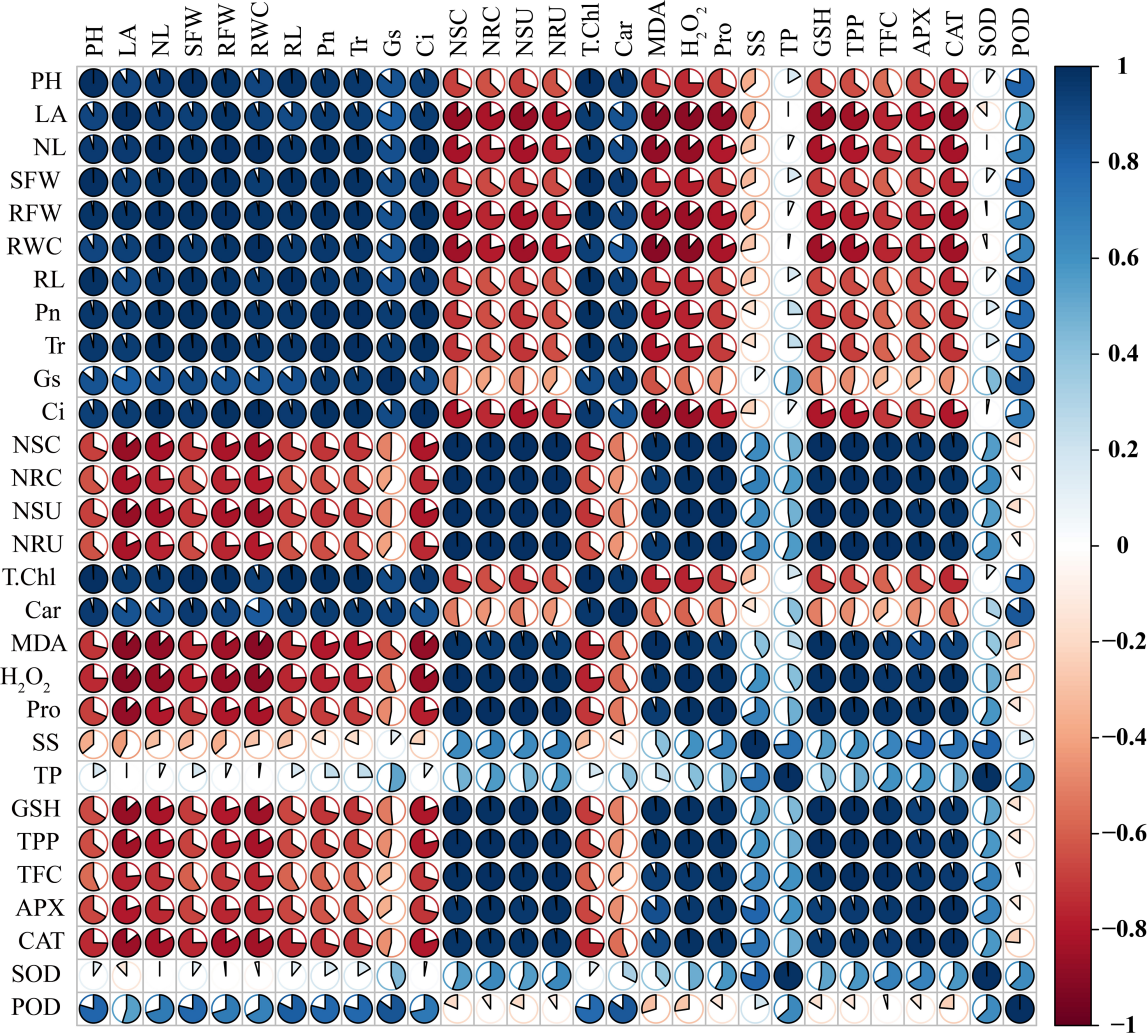
Pearson’s correlation analysis (*p*< 0.05) was measured between different traits of sweetpotato. PH, plant height; LA, leaf area; NL, number of leaves; SFW, shoot FW; RFW, root FW; RWC, relative water content; RL, root length; RV, root volume; Pn, photosynthetic assimilation; Tr, transpiration rate; Gs, stomatal conductance; Ci, Intercellular CO_2_; NSC, Ni shoot concentration; NRC, Ni root concentration; NSU, Ni shoot uptake; NRU, Ni root uptake; T. Chl, total chlorophyll; Car, carotenoids; MDA, malonaldehyde; H_2_O_2_, hydrogen peroxide; Pro, proline. SS, soluble sugars; TP, total proteins; GSH, reduced glutathione; TPP, total polyphenols; TFC, total flavonoids; APX, ascorbate peroxidase; CAT, catalase; SOD, superoxide dismutase; and POD, peroxidase.

Sweetpotato plants treated with higher levels of Ni showed a substantial decrease in the plant phenotypic and physiological traits that are highlighted by the negative correlation between phenotypic and physiological parameters (plant length, leaf number, leaf area, shoot and root FW, SDSI, RDSI, RWC, and all root morphological traits) and osmolytes, GSH, polyphenols, flavonoids, APX, and CAT (**Figure 11**). Similarly, Ni root and shoot concentration also exhibited a negative correlation with all the plant phenotypic parameters, demonstrating the deleterious effect of higher Ni treatment on the development and growth of the sweetpotato plant. The photosynthetic pigments and assimilation in leaves of sweetpotato also showed a negative correlation with osmolytes, GSH, polyphenols, flavonoids, APX, CAT, and Ni concentrations and uptake (**Figure 11**). In contrast, the phenotypic parameter presented a positive correlation with the photosynthetic pigments and assimilation, which means that a higher level of photosynthetic rate can increase the growth and biomass of the sweetpotato.

**Figure 12:**
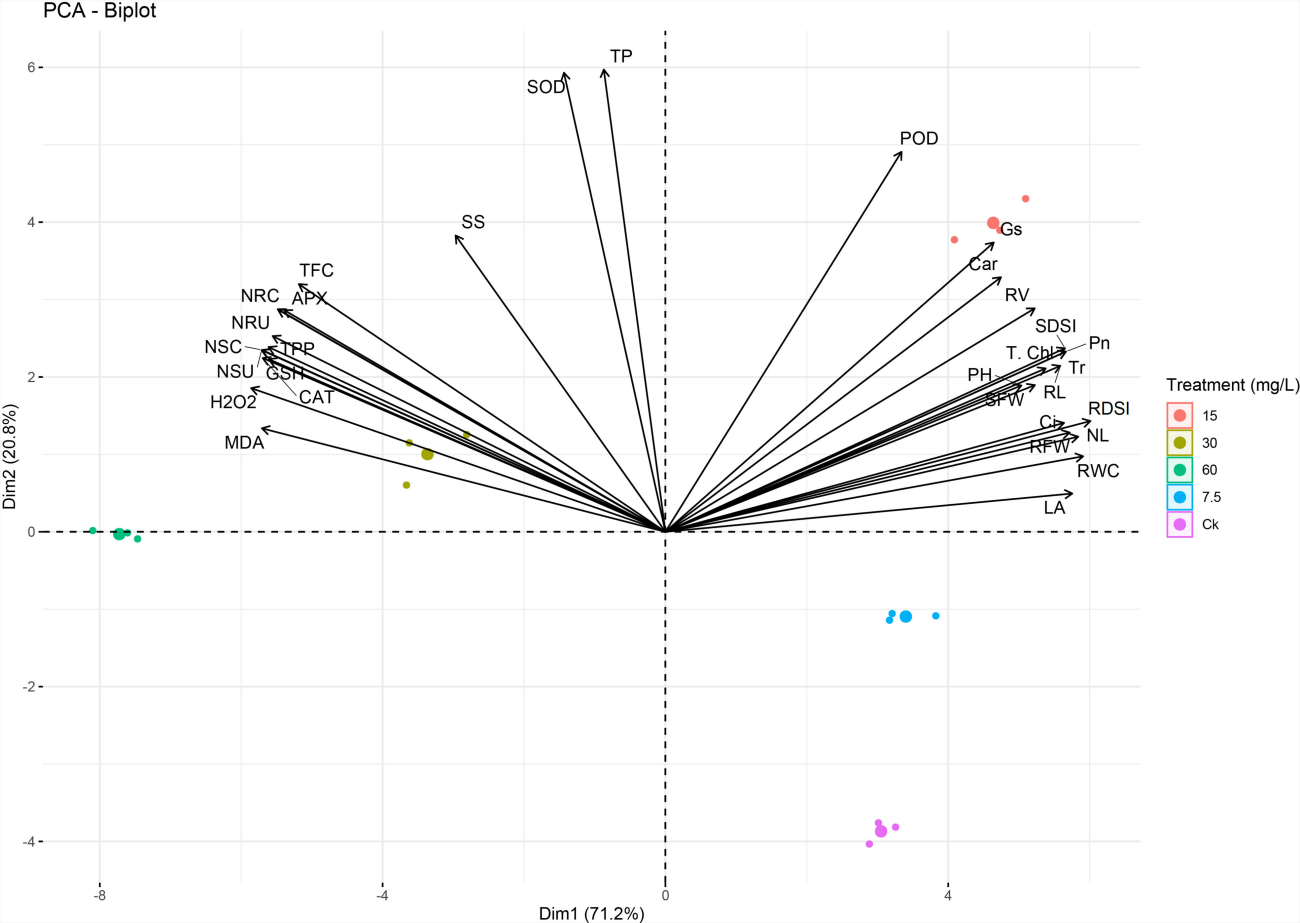
Principal component analysis (PCA) between phenotypic, physiological, and biochemical traits under different levels of Ni treatments in sweetpotato plant. MDA, malonaldehyde; H_2_O_2_, hydrogen peroxide; CAT, catalase; GSH, reduced glutathione; NSU, Ni shoot uptake; NSC, Ni shoot concentration; TPP, total polyphenols; NRU, Ni root uptake; NRC, Ni root concentration; APX, ascorbate peroxidase; TFC, total flavonoids; SS, soluble sugars; SOD, superoxide dismutase; TP, total proteins; POD, peroxidase; Gs, stomatal conductance; Car, carotenoids; RV, root volume; SDSI, shoot dry weight susceptibility index; T. Chl, total chlorophyll; Pn, photosynthetic assimilation; PH, plant height; Tr, transpiration rate; RL, root length; SFW, shoot FW; Ci, Intercellular CO_2_; NL, number of leaves; RDSI, root dry weight susceptibility index; RFW, root FW; RWC, relative water content; and LA, leaf area.

A PCA analysis showed clear deviations among phenotypic, physiological, and biochemical indices (**Figure 12**), which confirmed the trends of the results. The variables that exist closely and in the same quadrant are positively correlated. Black arrows correlate the studied parameters, while different color dots indicate different Ni treatments. A variance of 92% was observed for both principal components, and PCA1 was elucidated 71.2% and PCA2 20.8% for the mentioned plant phenotypic and physiological attributes. Ni treatment at a higher level showed a strong negative correlation of the growth, biomass, physiological traits, chlorophyll, and RWC with ROS, osmolytes, antioxidants, APX, CAT, SOD, and Ni concentration and uptake (**Figure 12**).

## Discussion

The low dose of Ni is required for enhanced plant growth and production and helps synthesize several biomolecules as their integral component. In a recent study, a low level of Ni was essential in minimizing the severity of biotic stress in plants (Barcelos et al., 2018). However, a higher level has a deleterious influence on plant growth and productivity (Gajewska and Skłodowska, 2008). Different studies reported a reduction in growth and biomass under Ni treatment (Singh et al., 2004; Amjad et al., 2020; Helaoui et al., 2020).

The current study demonstrated the effects of different concentrations of Ni in regulating the phenotypic, physiological, and biochemical responses of sweetpotato. This experiment revealed that the plant phenotypic parameters were enhanced at low levels of Ni treatment (7.5 and 15 mg L^-1^); however, higher concentrations (30 and 60 mg L^-1^) significantly hampered these phenotypic parameters. Similarly, the root morphological traits were also improved under low-level treatments (7.5 and 15 mg L^-1^), and a significant decrease was detected in higher-level Ni treatments. These findings are in agreement with the results in rice, wheat, indian mustard, and garden pea, which depicted increment of growth and biomass of roots and shoots under low dose of Ni and negative effect of Ni under higher dosage (Singh et al., 2004; Gajewska and Skłodowska, 2008; Rizwan et al., 2017; Kaur et al., 2019). In a recent study, a low level of Ni showed a critical role in minimizing the severity of powdery mildew in soybean plants (Barcelos et al., 2018). Therefore, based on these findings and literature, we suggest that using Ni on sweetpotato at a lower level is beneficial for growth and might help minimize the severity of mildew. Some other studies reported the reduction of growth and biomass in many plants under Ni stress (Amjad et al., 2020; Helaoui et al., 2020; Altaf et al., 2021; Aqeel et al., 2021). This negative effect of Ni at a higher level was probably due to interventions in several metabolic and biochemical processes, reduction in cell wall elasticity, plant cell proliferation, and disruption in mitotic cell division (Gajewska and Skłodowska, 2008; Yang et al., 2017). These molecular shifts can reason to stunted root and shoot biomass and growth. Previous literature revealed that heavy metal tolerance depends on the plant species and genotypes and might be organ-dependent.

In this study, we also found the same trend for transpiration rate, photosynthetic assimilation, and photosynthetic pigments, which increased at the low level of Ni treatments and reduced at the higher level of Ni treatment. Higher levels of Ni stress possibly decrease the photosynthetic activities and photosynthetic pigments of leaves by disturbing the membrane permeability, CO_2_ fixation, chloroplastic ultrastructures, and electron transport mechanisms. Based on these results, we suggest that less Ni translocation from roots to the above-ground parts might be linked with the reduced metal-induced transpiration rate (Yang and Tang, 2015; Abedini and Mohammadian, 2018). This reduced translocation subsequently caused a reduction in the growth, biomass, and development of the sweetpotato. Previous studies showed a reduction of growth and biomass in *Amaranthus paniculatus*, *Psidium guajava*, tomato, and mung bean also had lower transpiration rate, photosynthetic assimilation, and photosynthetic pigments under Ni stress (Bazihizina et al., 2015; Pietrini et al., 2015; Altaf et al., 2021; Aqeel et al., 2021).

According to Rizwan et al. (2017), rice shoots exposed to different Ni treatments responded similarly, and roots showed more uptake than the leaves. Similarly, Roccotiello et al. (2022) in tomato, Zhao et al. (2019) in lettuce, and Helaoui et al. (2020) in *Medicago sativa* showed an increment in the content of Ni in shoots and roots, and more content was present in roots than shoots. The present study also revealed a significant increase in Ni accumulation in the shoots and roots of sweetpotato with increasing Ni levels. Ni accumulated mainly in the sweetpotato roots, and comparatively lesser quantities were transported to the leaves. The distribution of Ni in tissues shows a variation in chemical form resulting from complexation with ligands produced by plants. The uptake of heavy metals from the roots can be passively transported to the shoots *via* the xylem vessels. The current study also revealed a minor translocation of Ni from the roots to the above-ground parts, indicating that the sweetpotato roots had largely retained Ni. This variance in the distribution of heavy metals in the roots and shoots of sweetpotato was a possible reason for different growth represses of these organs. A previous study observed that a reduction in the root-to-shoot length ratio was associated with an increment in the root-to-shoot ratio of the heavy metal accumulation in the plant (Gajewska and Skłodowska, 2008).

RWC is a convenient parameter to calculate plant abiotic stress. We found a decrease in the RWC under higher Ni treatments, exhibiting that sweetpotato plants were under stress. Previous studies displayed a decrease in RWC in the leaves under Ni stress (Gajewska and Skłodowska, 2008; Zaid et al., 2019; Jahan et al., 2020). It could be proposed that the sweetpotato under Ni stress might be associated with osmoprotective processes. Previous literature revealed the important role of osmolytes (proline and soluble sugar) in response to metal toxicity. Particularly proline, which could be strongly associated with its antioxidative properties and capability to protect the antioxidant enzymes, as well as act as a metal chelator (Uruç Parlak, 2016; Aqeel et al., 2021; Bashir et al., 2021). The present study showed an increase in the production of osmolytes under Ni treatments. Moreover, these osmolytes help guard plant cellular membranes and maintain turgor pressure that minimizes the harmful effects on plants.

Augmentation of ROS levels and lipid peroxidation in plant tissues is a critical response to stress environments (Kumar et al., 2020; Kumar et al., 2021b). An increase in MDA and H_2_O_2_ content in response to Ni stress was observed in the leaves of *Vicia sativa*, tomato, and rice (Ivanishchev and Abramova, 2015; Rizwan et al., 2017; Altaf et al., 2021). It has been proposed that H_2_O_2_ and MDA have a vital role in growth inhibition under heavy metal stress (Gajewska and Skłodowska, 2008; Amjad et al., 2020). H_2_O_2_ acts as a substrate for peroxidases, which have a role in cell wall stiffening that could ultimately constrain cell elongation. It can also negatively affect the proliferation of plant cells (Santoro et al., 2005). We also found an increment in MDA and H_2_O_2_ content in the leaves of sweetpotato with the increment of Ni stress, which could be involved in the growth inhibition of sweetpotato.

Antioxidant enzymes markedly reduce oxidative stress and ROS under different abiotic stresses (Sinha et al., 2005). In this study, we discovered that increasing the Ni application led to an increase in the APX and CAT activities in the leaves of sweetpotato.This showed that APX and CAT might help in the defense against H_2_O_2_ and help strengthen the plant cell wall. This study showed agreement with the results of Amjad et al. (2019) in maize, Amjad et al. (2020) in tomato, and Zhao et al. (2019) in lettuce, which showed the augmentation of both enzymes under Ni stress. In the present study, we found an increase in the activity of SOD and POD up to 15 mg L^-1,^ then decreased later, but the activities of both enzymes were still significantly higher than in the control plants. Application of a higher concentration of Ni reduced the activity of SOD and POD, which agree with the results of Pietrini et al. (2015) in *Amaranthus paniculatus*, Demirezen Yilmaz and Uruç Parlak (2011) in *Gronlendia densa*, and Zhao et al. (2019) in lettuce. In contrast, when exposed to Ni stress, a significant increase or decrease in SOD and POD activity was observed in wheat, rice, and maize (Gajewska and Skłodowska, 2008; Rizwan et al., 2017; Amjad et al., 2019). Inconsistent results regarding the response to Ni toxicity were not only observed in the case of POD and SOD but also other studied antioxidant enzymes (CAT and APX). The variances may elucidate these differences between the activities of enzymes in Ni tolerance in plant species, developmental stages, dosage of heavy metals, duration of the experiment, and other variations in experimental conditions. This increase in overall enzymatic activities seems to be a typical antioxidative response of the sweetpotato plant.

Total proteins act as osmotin and play a vital role in tolerance against abiotic stresses (Qados, 2011; Kumar et al., 2021b). The present study depicted the increase of total protein contents up to 15 mg L^-1^ Ni treatment, then it started to decrease. According to Rajendran et al. (2019), heavy metal treatment has synthesized new polypeptides, which can help increase heavy metal tolerance. Previous studies by Demirezen Yilmaz and Uruç Parlak (2011) and Helaoui et al. (2020) showed an increment in the protein content under low Ni stress; however, many other studies showed a reduction in total proteins under Ni stress (Rizwan et al., 2017; Rizwan et al., 2018; Hassan et al., 2019). This decrease in protein content at higher levels might be due to the higher production of ROS that can damage proteins, membrane lipids, pigments, and nucleic acids (Demirezen Yilmaz and Uruç Parlak, 2011). GSH increased under Ni stress in this study. Antioxidant GSH helps plants detoxify free radicals and scavenge ROS (Kumar et al., 2021b). Different studies also reported the increment of GSH in the shoots under Ni stress (Rizwan et al., 2017; Rizwan et al., 2018). Since GSH is a precursor of phytochelatins and phytochelatins could play a role in tolerance and detoxification by chelating metals, Ni-stressed plants exhibit phytochelatin biosynthesis (Hall, 2002; Rizwan et al., 2017).

Secondary metabolites (total polyphenols and flavonoids) can act as a second-line defense and enhance the enzyme activities in stress conditions by scavenging free radicals (Kofroňová et al., 2020; Kumar et al., 2020). Furthermore, phenols have the potential for chelating metals, which restrict the excessive Ni by catching the hydroxyl radical (Jahan et al., 2020). This study found a significant increment in polyphenols and flavonoid content under Ni stress. The previous studies by Jahan et al. (2020) and Strejckova et al. (2019) also showed an increment of polyphenols and flavonoids under Ni stress. This increase in polyphenols could be due to the increment in phenolic compounds, including gallic acid, rutin, salicylic acid, vanillic acid, quercetin, coumaric acid etc. (Sarker and Oba, 2018; Kumar et al., 2020). Another reason might be the increase in the activity of different enzymes of phenylpropanoid pathways (André et al., 2009; Kisa et al., 2019). This induced accumulation of phenols in sweetpotato plants could help in tolerance against Ni toxicity, but further studies are required for confirmation.

## Conclusion

Ni toxicity is a concerning point for sweetpotato growers. Results showed that higher treatments substantially reduced growth, biomass, and root morphological traits. Ni toxicity causes oxidative injuries as a stubborn increase of H_2_O_2_ and MDA and reduced RWC, gas exchange, and photosynthetic pigment. The present study determined that the sweetpotato plant can tolerate Ni treatment up to 15 mg L^-1^ by reducing oxidative stress. It is also depicted that increased activities of highlighted osmolytes, antioxidants, and enzymes are insufficient to regulate the higher Ni toxicity. We also propose that using low Ni-contaminated soil could be helpful for improving sweetpotato growth and as a phytoremediator in moderate Ni-contaminated soil. Different soil amendments or using different chemicals for foliar applications to reduce Ni toxicity and increase the growth and production of sweetpotato.

## Data availability statement

The original contributions presented in the study are included in the article/**Supplementary Materials**. Further inquiries can be directed to the corresponding authors.

## Author contributions

Conceptualization: GZ, YC, and SK. Funding acquisition: GZ. Data curation, investigation, and writing—original draft: SK. Validation: SK and SJ. Methodology: SK, and MW. Writing—review and editing: SK, SF, AQ, and YL. All authors contributed to the article and approved the submitted version.

## Funding

This work was supported by the earmarked fund for CARS-10-Sweetpotato.

## Acknowledgments

The authors acknowledge all the staff members of the Laboratory of Analytical & Testing Center, Hainan University, for providing technical support and help during the experimental work.

## Conflict of interest

The authors declare that the research was conducted in the absence of any commercial or financial relationships that could be construed as a potential conflict of interest.

## Publisher’s note

All claims expressed in this article are solely those of the authors and do not necessarily represent those of their affiliated organizations, or those of the publisher, the editors and the reviewers. Any product that may be evaluated in this article, or claim that may be made by its manufacturer, is not guaranteed or endorsed by the publisher.
